# Diagnosing Autism in women - the gender based complexities of assesment and support

**DOI:** 10.1192/j.eurpsy.2026.11504

**Published:** 2026-07-31

**Authors:** J. Rausch

**Affiliations:** Department of Psychiatry and Psychotherapy, University of Freiburg, Faculty of Medicine, Freiburg, Germany

## Abstract

**Introduction:**

While the focus of research and diagnostics has shifted from non-verbal, low-IQ Autism to high-functioning Autism it has become more apparent that there is a discrepany between the number of males and females being diagnosed. These findings are not only presented in epidemological studies but in studies which attempt to actively identify cases in the general population as well. This leads to the question whether there is an inherent difference in the prevalence or if we are not as good as detecting Autism in females and if so, why that might be. This talk will give an overview of the research already conducted in this area as well as concerete examples from years of Autism diagnostics.

- *This talk was already accepted for the EPA 2025, however, due to a freshly broken leg unfortunatly I had to excuse myself and be absent from the congress. Therefore I would like to submit this topic again. If this is not possible due to my absence last year or because the topic is not of interest two years in a row, I fully understand. -*

**Objectives:**

The aim of this presentation is to increase awareness of the presentation of symptoms of Autism in women and the real life implications in the diagnostic process.

**Methods:**

This presentation will conclude from the statistical research drawn from various sources, such as Global Burden of Disease study published in the Lancet and the recent numbers by the CDC for a broad epidemiological background and shift to the real life implications in the day to day life in a diagnostic centre for Autism.

**Results:**

According to the Global Burden of Disease Study 2021 the prevelance of Autism in females is 0.5% to the male 1.1%. However, in experienced clinical settings, the number often shifts even further toward the male population. Since female presentation tends to be overlooked, this talk aims to focus on the differences in the gender presentation.

**Conclusions:**

Through this talk hopefully the perspective of judging symptoms according to their gender presenation should become clearer.

**Disclosure of Interest:**

None Declared

